# Bimorph piezoelectric vibration energy harvester with flexible 3D meshed-core structure for low frequency vibration

**DOI:** 10.1080/14686996.2018.1508985

**Published:** 2018-09-25

**Authors:** Takuya Tsukamoto, Yohei Umino, Sachie Shiomi, Kou Yamada, Takaaki Suzuki

**Affiliations:** a Division of Mechanical Science and Technology, Gunma University, Kiryu-shi, Gunma, Japan; b Japan Science and Technology Agency, PRESTO, Kawaguchi, Saitama, Japan

**Keywords:** Vibration energy harvester, piezoelectric, PVDF, piezoelectric coupling analysis, low frequency, 50 Energy materials, 202 Dielectrics/Piezoelectrics/Insulators, 206 Energy conversion/transport/storage/recovery

## Abstract

This paper proposes a bimorph piezoelectric vibration energy harvester (PVEH) with a flexible 3D meshed-core elastic layer for improving the output power while lowering the resonance frequency. Owing to the high void ratio of the 3D meshed-core structure, the bending stiffness of the cantilever can be lowered. Thus, the deflection of the harvester and the strain in the piezoelectric layer increase. According to vibration tests, the resonance frequency is 15.8% lower and the output power is 68% higher than in the conventional solid-core PVEH. Compared to the solid-core PVEH, the proposed meshed-core PVEH (10 mm × 20 mm × 280 μm) has 1.3 times larger tip deflection and the maximum output power is 24.6 μW under resonance condition at 18.7 Hz and 0.2G acceleration. Hence it can be used as a power supply for low-power-consumption sensor nodes in wireless sensor networks.

## Graphical Abstract

**Figure:**
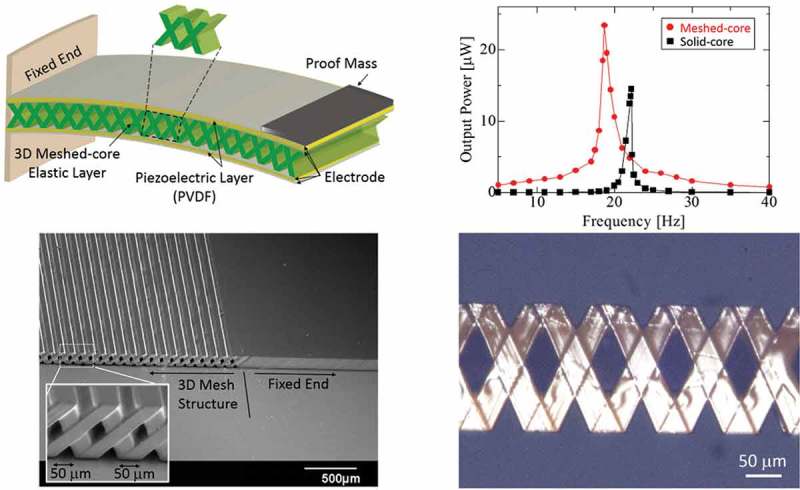

## Introduction

With the growing performance of low-power-consumption devices and the rapid development of Internet of Things (IoT) communication technology, it is said that the era of trillion sensors is arriving. Meanwhile, the power supply required for the enormous number of sensor nodes is a major issue [1–3]. Thus, as a battery for self-powered sensor, energy harvesting has attracted much research attention. Energy harvesting is one of the powering technologies that obtains electric power from external small energy sources, and is expected to omit the need for replacing and charging conventional button batteries. Light, heat, radio waves, and vibration have been extensively studied as sources of energy harvesting. Among them, vibration has been drawing considerable attention because vibration energy has relatively high energy density, and is independent of the operating environment and weather [4]. Although many vibration energy harvesters have been studied, most of them operate at relatively high external frequencies (above 100 Hz) [5,6]. They are inefficient at low-frequency (under 20 Hz) or low-acceleration (0.1 to 1 G) conditions which relate to various human activities, bridge vibration, and house appliances. Some studied harvesters can operate at low frequencies, but they are either too large to be wearable, or their electrical output is insufficient for powering typical consumption devices [7–9].

Vibration energy harvesting is categorized into three types: electrostatic, electromagnetic, and piezoelectric. Piezoelectric vibration energy harvesters (PVEHs) exhibit (1) simple configuration, (2) high output voltage, and (3) high compatibility with microfabrication technology [10,11]. Many unimorph cantilever-type harvesters (i.e. consisting of a piezoelectric film and an elastic layer to support the piezoelectric film and to control vibration characteristics) have been proposed for PVEHs [12–16]. Because the configuration of a unimorph cantilever is simple and the fabrication process is relatively easy, many unimorph harvesters are fabricated using piezoelectric materials such as lead zirconate titanate (PZT) and polyvinylidene fluoride (PVDF). However, their output power is low because there is only one piezoelectric layer. Moreover, to harvest electric energy from low-frequency band vibration, it is necessary to select a flexible material for the elastic layer so as to lower the resonance frequency, because the types of the piezoelectric materials are limited. However, if the elastic layer becomes flexible, the neutral axis is shifted from the elastic layer side toward the piezoelectric layer side. This shift reduces both the output power and the strain generated in the piezoelectric layer. Therefore, it is difficult to achieve both low resonance frequency and high output power in a unimorph cantilever-type vibration energy harvester.

On the other hand, in a bimorph cantilever-type vibration energy harvester [17,18], the elastic layer is sandwiched between two piezoelectric layers. Unlike in the unimorph cantilever, in the bimorph type, the neutral axis is usually at the center of the cantilever regardless of the Young’s modulus and thickness of both piezoelectric and elastic layers. Therefore, by increasing the film thickness of the elastic layer, the distance between the neutral axis and the piezoelectric film can be increased. Hence, the amount of strain in the piezoelectric layers increases under certain deflection, and consequently, the amount of generated power increases as compared to the unimorph type. In addition, several methods have been reported for increasing the output power by optimizing the cantilever shape and thickness of each layer have been reported [19–21]. Hence, the bimorph configuration is effective in improving the amount of output power. However, along with an increase in the elastic layer’s thickness and optimization of the shape for power improvement, the resonance frequency also increases. Additionally, the resonance frequency increases with the miniaturization of the device size. Hence, it is difficult to achieve both improvements in output power and reduction in resonance frequency.

Therefore, in this study, we fabricate a bimorph cantilever-type PVEH incorporating a flexible 3D meshed-core elastic layer. The flexible 3D meshed-core elastic layer can lower the bending stiffness and increase the cantilever deflection while maintaining the distance between the neutral axis and the piezoelectric layer. This approach can lower the resonance frequency while improving the output power without any change in the composed material, device size, and basic fabrication process.

## Overall design of PVEH

### Configuration and structure

**Figure 1.**
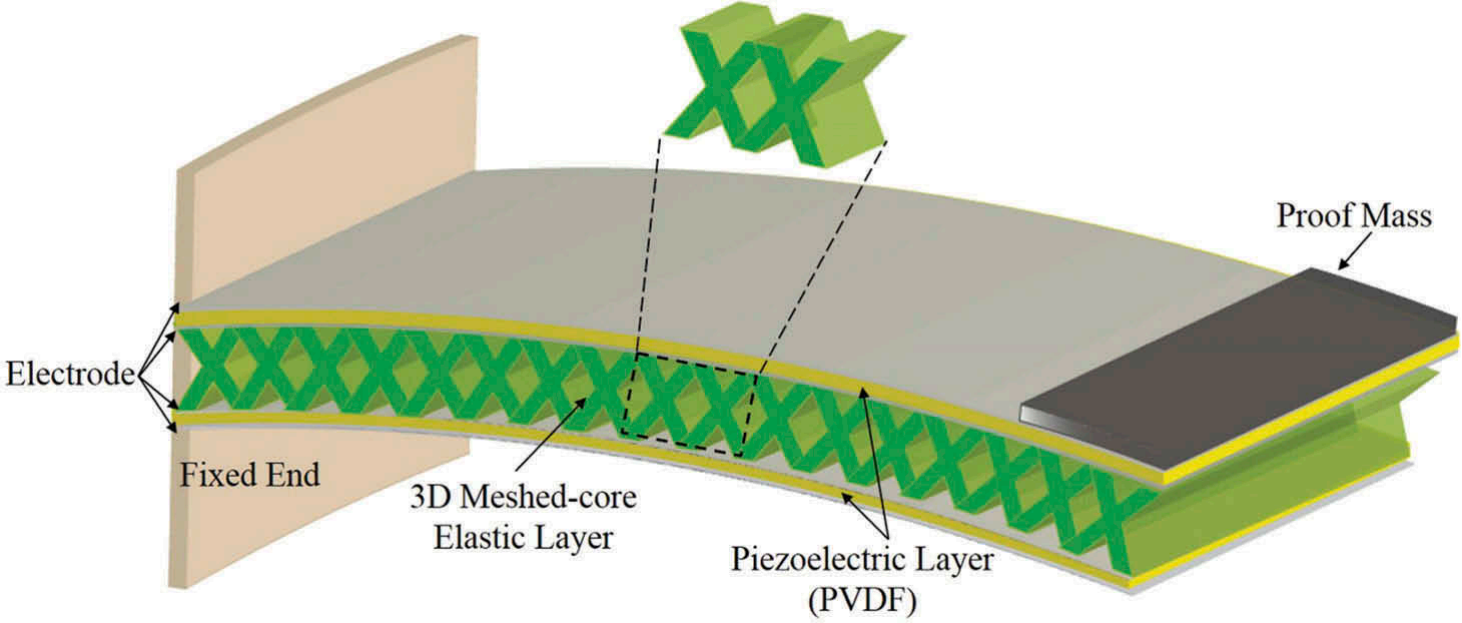
Schematic of the proposed PVEH with 3D meshed-core structure (10 mm × 21 mm × 280 μm).

The proposed meshed-core PVEH is a bimorph-type cantilever consisting of the electrodes, two piezoelectric layers, and an elastic layer sandwiched between the piezoelectric layers. The elastic layer is formed of a thick-film photoresist and has a 3D meshed-core structure to lower resonance frequency, as shown in Figure 1. The conventional studies to lower resonance frequency use the methods as follows: (1) low Young’s modulus of polymer materials [7], (2) heavy weight of the proof mass [13], (3) long entire length of the cantilever (from the fixed end to the free end) [8,9,16]. The simplest approach is to use heavy proof mass; however, the mass and volume of the whole device will easily increase and it is not preferable for practical applications.

So we propose the meshed-core structure as an elastic layer among the various possibilities of artificial structures for the following reasons: (1) the meshed-core structure is suitable to make a cantilever flexible by having voids in three dimensions, so that the second moment of area tends to be smaller compared to a two-dimensional periodic structure (e.g. square lattice). (2) The meshed-core structure can be combined with previously studied structures and shapes such as ‘2D structures (e.g. meandered or spiral structure [9,16])’ for reducing the resonance frequency, and 2D shapes (e.g. trapezoid, triangle [20,21]) for improving power generation. (3) The design and the fabrication process to make a complex 3D mesh structure are relatively simple when using 3D inclined exposure. Since UV light is applied vertically to the substrate in general 2D photolithography, the manufacturable structures are limited to 2D shapes. Thus, no research has yet considered to fabricate meshed-core structure and to adopt it to vibration energy harvester for lowering resonance frequency because of this limitation. For the above reasons, we adopted the meshed-core structure as the elastic layer to lower the resonance frequency and improve the output power without any change in the composed material, device size, and basic fabrication process.

The meshed-core structure has voids, and the flexibility of the elastic layer can be controlled by adjusting the volume void fraction. In other words, the meshed-core structure can reduce the bending stiffness of the bimorph PVEH, and thus, increase the deflection of the cantilever. As the deflection of the bimorph PVEH increases, the amount of the strain occurring in the piezoelectric layer increases. Because the strain is proportional to the output voltage, reducing the bending stiffness improves the amount of power generated. Therefore, this method aims at improving the power generation amount and reducing the resonance frequency without changing the composed material, device size, and basic fabrication process. The meshed-core structure can be fabricated using the inclined exposure method [22]: exposing ultraviolet light twice to the substrate from oblique directions. The process is detailed in the next chapter.

Regarding the two piezoelectric layers, PVDF films, which are formed of a polymer piezoelectric material, are used. Although PVDF has an inferior piezoelectric constant compared to ceramics’ inorganic piezoelectric materials such as PZT and barium titanate [21,23], it enables large deformation and exhibits excellent flexibility [24]. Since the materials used in the proposed PVEH, except for the electrodes, are made of polymer materials, the proposed bimorph cantilever is flexible. Thus, the resonance frequency can be lowered and the proposed PVEH can generate output power efficiently at low frequencies.

In the bimorph configuration, the neutral axis is at the center of the cross section of a beam, and by increasing the film thickness of the elastic layer, the distance between the piezoelectric film and the neutral axis can be easily maintained. Here, as the distance from the piezoelectric film to the neutral axis is proportional to the output voltage, it can be expected to generate high output power. However, increasing the film thickness of the elastic layer also increases the bending stiffness of the bimorph cantilever, and it becomes difficult to achieve both low resonance frequency and high output power. Therefore, in this study, we investigate the effects of the meshed-core structure on lowering the bending stiffness and improving the output power simultaneously.

### FEM structural analysis

**Figure 2.**
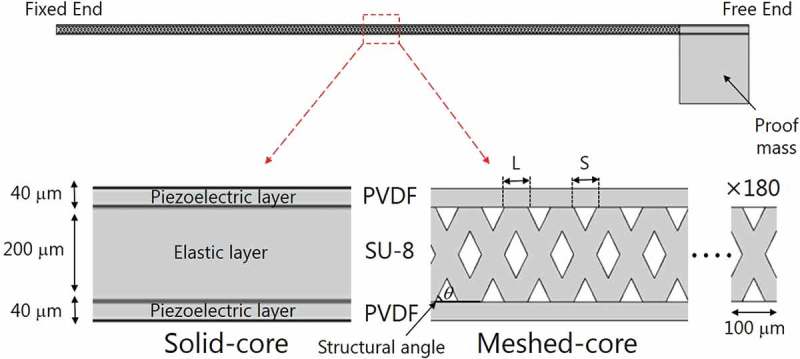
Schematic of a FEM structural analysis model. In the meshed-core structure, the line length *L* and line spacing *S* satisfy the relation *L* + *S* = 100 μm.

**Figure 3.**
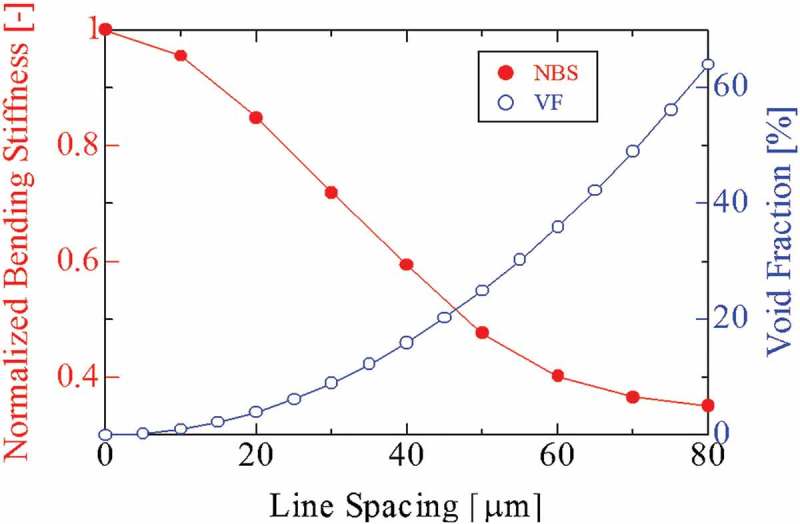
Volume void fraction and normalized bending stiffness as a function of line spacing of the meshed-core structure. The normalized bending stiffness is calculated by dividing the bending stiffness of a harvester with the meshed-core elastic layer by that of a harvester with a solid-core elastic layer.

To verify the validity of the proposed meshed-core PVEH, we compare the conventional solid-core type and the meshed-core type by structural analysis. The analysis is performed using finite element method (FEM) software COMSOL Multiphysics 5.2a. The analytical model is shown in Figure 2, and the dimensions and material properties used in the FEM analysis are listed in Tables 1 and 2. The analytical model comprises three layers: two piezoelectric layers and an elastic layer. Although the electrodes are deposited on both sides of two piezoelectric films in an actual bimorph harvester, the influence on the analysis results can be ignored because the electrode thickness is sufficiently thin (50 nm) compared to each piezoelectric film of 40 μm thickness and elastic layer of 200 μm thickness. The size of the bimorph cantilever is 10 mm in width × 20 mm in length × 280 μm in thickness. As for the meshed-core elastic layer in the bimorph cantilever, the line spacing and line length as shown in Figure 2 vary in the range of 10–90 μm, with the line length increasing in increments of 10 μm, and the sum of the line spacing and line length is designed to be 100 μm. One end of the bimorph cantilever is fixed and the other end is free. When a tip load is applied to the analytical model, the tip deflection under the boundary condition is calculated. Based on the calculated tip deflections, we calculate the ratio of the deflection amounts of the solid-core type and the meshed-core type. Because the tip deflection is inversely proportional to the bending stiffness, we normalize it by the tip deflection of the solid-core bimorph cantilever and the meshed-core type. Figure 3 presents the results of FEM structural analysis. It shows how much the bending stiffness of the meshed-core structure can be reduced in the proposed PVEH. The horizontal axis of the graph represents the line spacing of the meshed-core structure. The left vertical axis represents the calculated normalized bending stiffness: The normalized bending stiffness is calculated by dividing the bending stiffness of the meshed-core bimorph cantilever by that of the solid-core type. The right vertical axis is a volume void fraction, which is expressed as *P_v_* and can be calculated by Equation (1). The volume void fraction is independent of the structural angle of the meshed-core structure and determined by the line spacing *S* and line length *L*.

(1) $$P_{v}=\frac{S^{2}tan\theta }{{\left(L+S\right)}^{2}tan\theta }=\frac{S^{2}}{{\left(L+S\right)}^{2}}$$

**Table 1.** Dimensions of the proposed PVEH. The subscripts *p* and *e* indicate a piezoelectric material and an elastic layer, respectively.

Parameters	Values
Width	10 mm
Length	21 mm
Thickness: *t_p_*	40 μm
Thickness: *t_e_*	200 μm
Proof mass	1.0 g

**Table 2.** Parameters of FEM analysis.

Parameters	Values
Young’s modulus: *Y_p_*	0.95 GPa
Young’s modulus: *Y_e_*	3.25 GPa
Piezoelectric constant *d_31_*	22 pC/N
Piezoelectric constant *d_33_*	35 pC/N
Relative dielectric constant	12 [–]

The structural FEM analysis confirms that the bending stiffness of the bimorph-type cantilever is decreased by incorporating the meshed-core structure and changing the line spacing. The bending stiffness and resonance frequency can be related using Equation (2) [13], and the resonance frequency can be lowered by reducing the bending stiffness.

(2) $$f_{n}=\frac{1}{2\pi }\sqrt{\frac{3EI}{\left(m+\frac{33}{140}m_{s}\right)l^{3}}}$$

where *f_n_* is the resonance frequency of the cantilever, *E* is Young’s modulus, *I* is the second moment of inertia, *m* is the mass of the cantilever, *m_s_* is the tip proof mass, and *l* is the length of the cantilever from the fixed end to free end. From the result of the structural analysis, we conclude that in the meshed-core PVEH the bending stiffness and resonance frequency can be controlled by adjusting the line spacing and hence the volume void fraction.

### FEM piezoelectric coupling analysis

**Figure 4.**
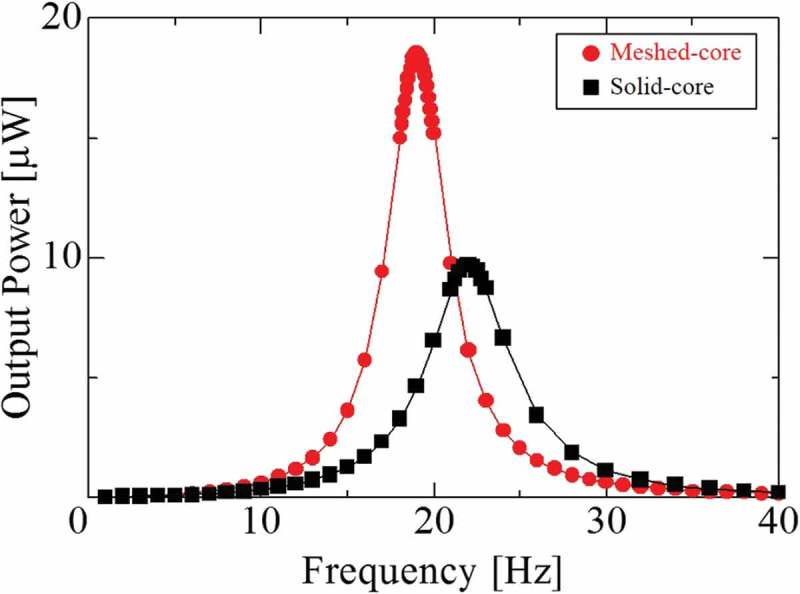
FEM result of maximum output power as a function of vibration frequency under each optimum load resistance (meshed-core 17 MΩ, solid-core 13 MΩ), and 0.2 G acceleration.

To evaluate the power generation performance of the meshed- and solid-core PVEHs, we perform piezoelectric coupling analysis using COMSOL Multiphysics 5.2a. Two layers of the PVDF piezoelectric film are electrically connected in series, and the power consumption at the load resistance is evaluated. Here, each load resistance used in both piezoelectric coupling models is the optimum and the resistance values are 13 MΩ for the meshed-core type and 11 MΩ for the solid-core type. These optimum load resistances are calculated in advance by evaluating the maximum output power with changing the load resistance under resonance frequency. Figure 4 shows the results of frequency response analysis in the range of 1–40 Hz. The resonance frequency of the meshed-core PVEH is 18.0 Hz and that of the solid-core type is 22.1 Hz. The meshed-core type has 18.6% lower resonance frequency and a 74.5% higher output power. This is because the bending stiffness of the meshed-core PVEH is reduced due to the introduction of a 3D meshed-core structure with voids into the elastic layer and the resonance frequency is decreased. Simultaneously, the amount of tip deflection of the bimorph cantilever is increased. Therefore, the effectiveness of the meshed-core PVEH is confirmed by the piezoelectric coupling analysis and structural analysis.

## Materials and methods

### Fabrication process

**Figure 5.**
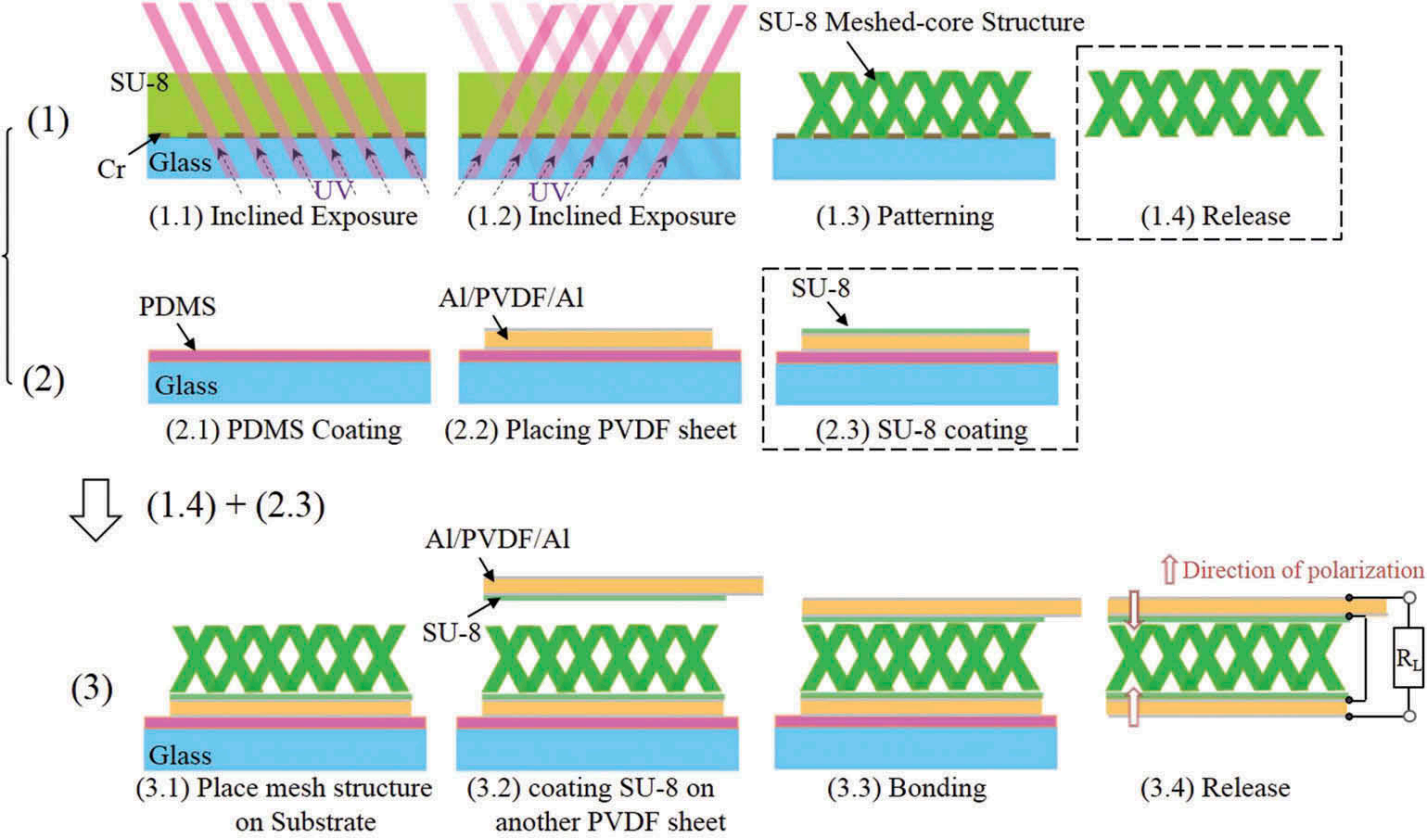
Fabrication process of bimorph meshed-core harvester.

**Figure 6.**
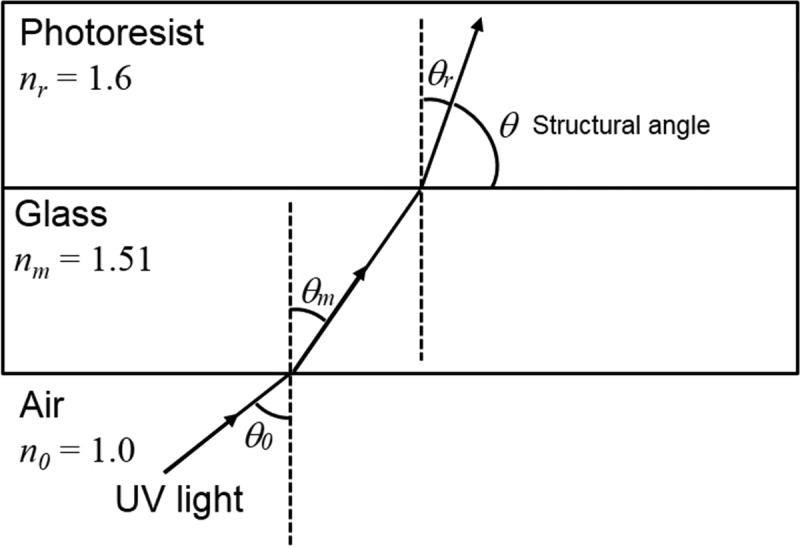
Optical path regarding the inclined exposure.

**Figure 7.**
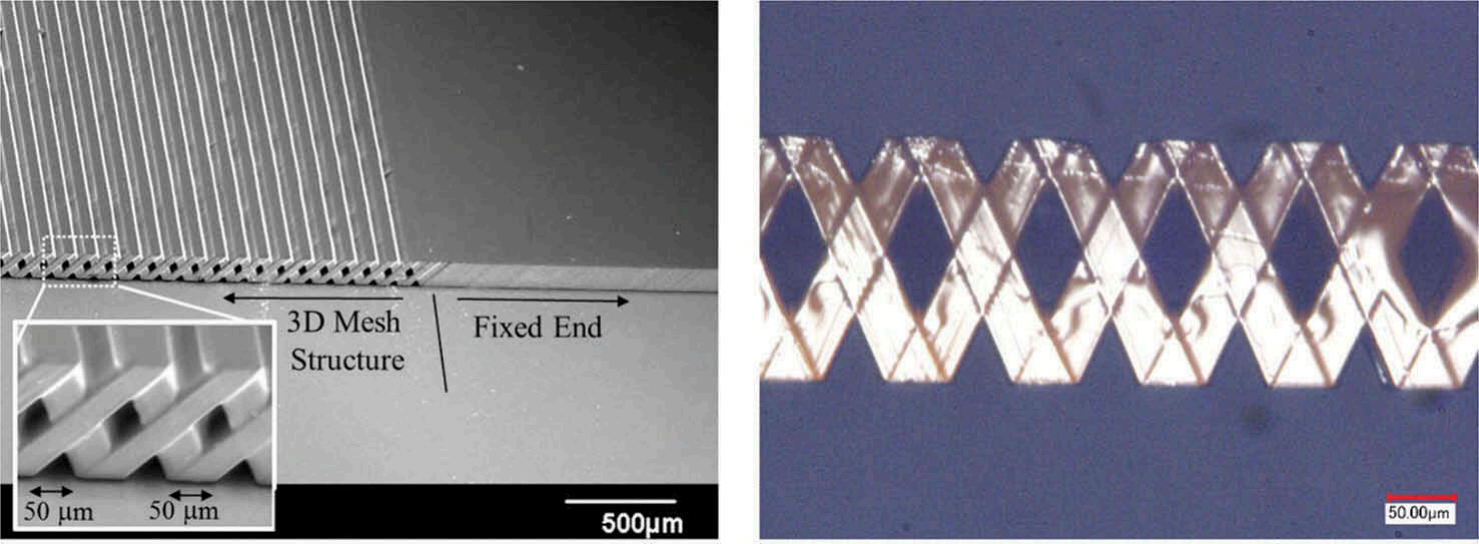
(a) SEM image of SU-8 meshed-core elastic layer and (b) optical image of 3D meshed-core structure.

The proposed PVEH comprises an SU-8 (SU-8 3005; Nihon Kayaku, Tokyo, Japan) elastic layer having a meshed-core structure and two PVDF piezoelectric layers (KF Piezo Film; Kureha Corporation, Tokyo, Japan) with Al electrodes. First, we develop a Cr pattern on a glass substrate for 3D meshed-core structure patterning. Then, acrylic resin (Shin-Etsu Chemical Corporation: barrier coat No.6) is coated on the Cr pattern as a sacrificial layer and baked at 95 °C for 10 min on a hot plate. After that, a 200-µm thick SU-8 layer is deposited by a spray-coating method, which can produce a thick film with a higher accuracy of thickness and better uniformity compared to the conventional spin coating method [25]. To evaporate the solvent of SU-8 used by the spray coater, the sample is baked on a hot plate at 95 °C for about 6 h. After sufficiently drying the SU-8 thick film, back-side-inclined UV exposure [22] is performed twice from different directions (wavelength: 365 nm, intensity: 150 mJ/cm^2^ × 2), as shown in Figure 5. The structural angle formed upon inclined exposure is given by

(3) $$n_{0}\sin\theta_{0}=n_{m}\sin\theta_{m}=n_{r}\sin\theta_{r}$$

(4) $$\begin{array}{c} \theta_{r}=\sin^{-1}\left(\frac{n}{n_{r}}\sin\theta_{0}\right) \end{array}$$

where *n_0_* is the refractive index of the atmosphere, *θ_0_* is the incident angle of the light, *n_m_* is the refractive index of the photomask, *θ_m_* is the refracting angle of the light in the glass, *n_r_* is the refractive index of the photoresist, *θ_r_* is the angle of the inclined structure, and *θ* is the structural angle as shown in Figure 6. Here, the refractive index of SU-8 is 1.6, so an incident angle of 53.1° is applied for inclined exposure. After developing SU-8 and forming the meshed-core pattern, it is immersed in a toluene solution and the meshed-core elastic layer is released by dissolving the sacrificial layer of the acrylic resin. Figure 7(a) shows a scanning electron microscopy (SEM) image of the fabricated 3D meshed-core elastic layer. From the SEM image, it is confirmed that both line length and line spacing are 50 μm, and a hollow structure is fabricated. Figure 7(b) shows an optical image obtained by observing a side surface of the meshed-core structure with a digital microscope. We confirm that the 3D structure is successfully fabricated as designed.

Next, polydimethylsiloxane is spin-coated as an adhesion layer on another glass substrate at 3000 rpm, and a PVDF film cut to the beam size is attached to it. A 50 nm Al layer is vapor-deposited on both sides of the PVDF film. A solution of SU-8 3005 diluted with a thinner is spin-coated on the PVDF film at 4000 rpm as an adhesion layer between the PVDF film and the SU-8 elastic layer.

**Figure 8.**
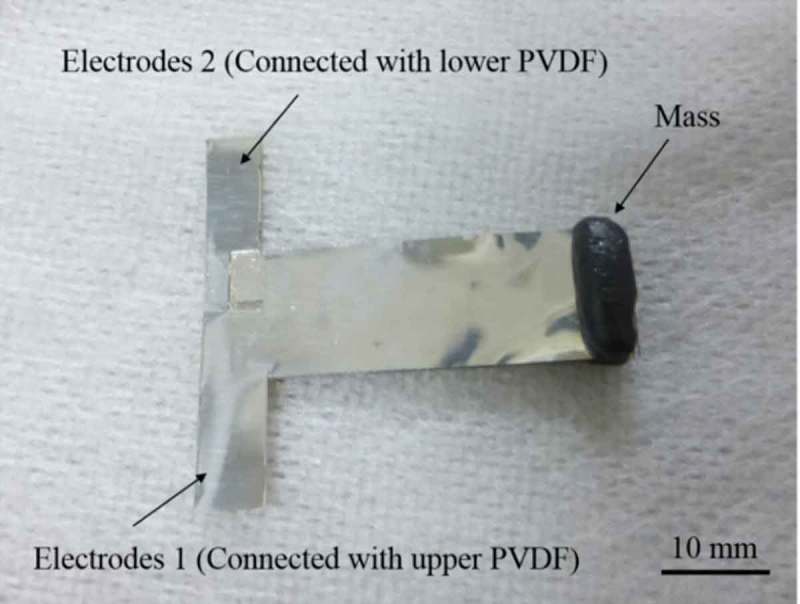
Photograph of fabricated bimorph meshed-core vibration energy harvester.

Finally, the bonding process is conducted. The fabricated meshed-core elastic layer is laminated on the PVDF film and the second PVDF coated with an SU-8 adhesion layer is bonded to the elastic layer thereon. The PVEH fabricated by the above process is air-dried for about 12 h. The reason for air-drying without increasing the temperature is that the PVDF film deteriorates the piezoelectric performance after heating to 80 °C [26]. Figure 8 shows the fabricated PVEH, which has two electrode nodes.

### Evaluation of PVEH

**Figure 9.**
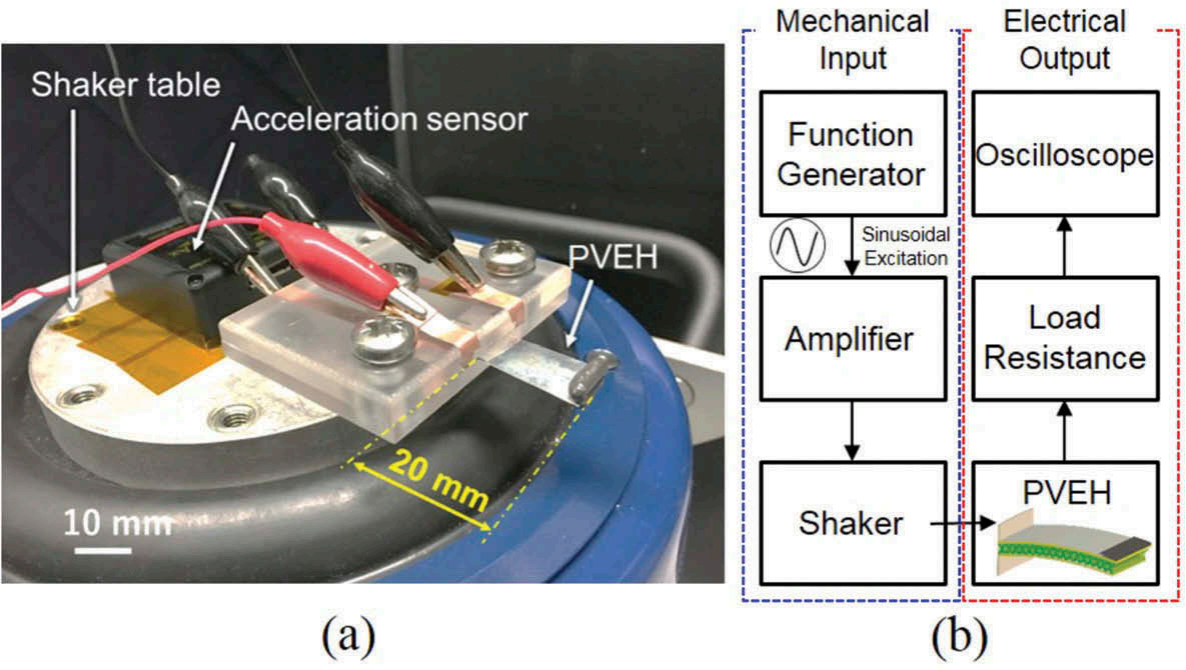
(a) Experimental setup and (b) evaluation system for PVEH.

To evaluate the power generation performance of the fabricated PVEHs, vibration tests are performed, in which the meshed- and the solid-core PVEHs are used to evaluate the output power, resonance frequency, and optimum load resistance. Here, both PVEHs are fabricated similarly and the occupied effective area of the piezoelectric films and the elastic layer and the film thickness of each layer are same. Note that only the internal structure of the elastic layer is different. Figure 9 shows the experimental setup and the system for evaluating PVEH performance. As for the fabricated PVEHs, two piezoelectric PVDF layers in the bimorph are connected in series. The power generation amount *P* is given by Equation (5), and calculated from the output voltage *V* and load resistance *R_L_*.

(5) $$P=\frac{V^{2}}{R_{L}}$$

To investigate the optimum load resistance value, the load resistance value is changed from 1 to 31 MΩ under the resonance condition. The acceleration used in the vibration test is 0.2 G (1.96 m/s^2^), which is equivalent to the vibration acceleration of human walking. The attached acceleration sensor on the shaker table is set to keep the vibration acceleration constant to 0.2 G when the frequency changes. The output voltage is evaluated while focusing on the relations among the internal resistance value of the piezoelectric material, load resistance, and input impedance of the oscilloscope.

## Results and discussion

**Figure 10.**
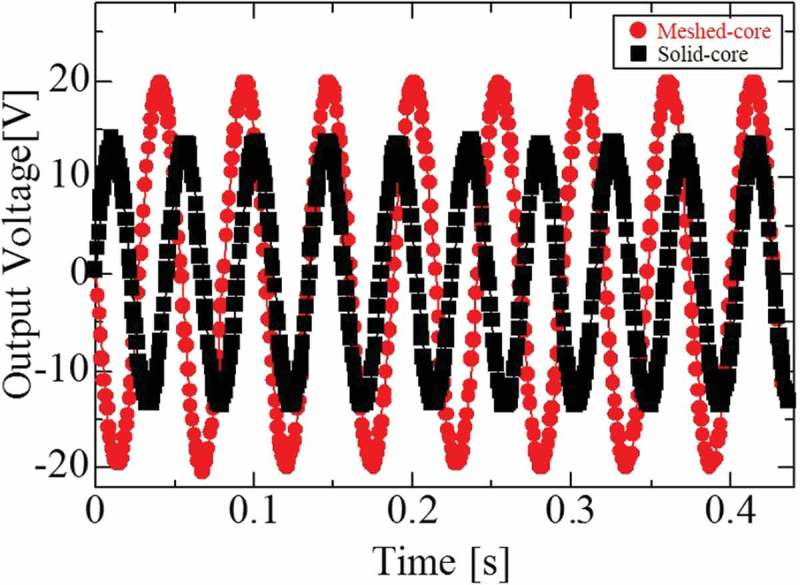
Sinusoidal measured voltage of load resistance under each resonance condition (meshed-core 18.7 Hz, solid-core 22.2 Hz), optimum load resistance (meshed-core 17 MΩ, solid-core 13 MΩ), and 0.2 G acceleration.

Figure 10 shows the output voltage waveform of the fabricated PVEHs under resonance condition. In both meshed- and solid-core PVEHs, the measured output voltage of load resistance shows a sinusoidal wave for the input sinusoidal excitation. The frequency of the output voltage coincides with that of the input sinusoidal waveform. In the meshed-core PVEH, the output voltage is 42.6% higher than that of the solid-core type. In addition, the maximum output voltage is 20.4 V for the meshed-core PVEH and 14.1 V for the solid-core type. The output voltage is measured under the resonance condition with optimum load resistance and the 0.2 G excitation acceleration.

**Figure 11.**
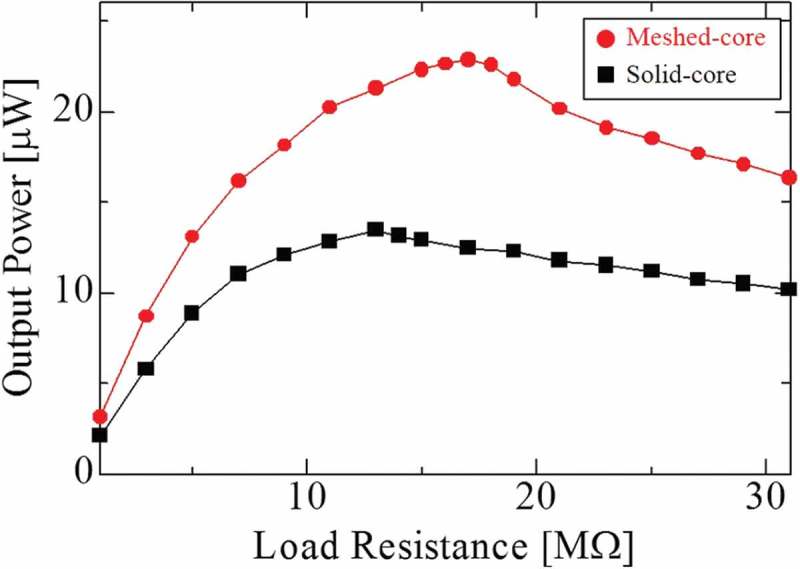
Maximum output power as a function of load resistance under each resonance condition (meshed-core 18.7 Hz, solid-core 22.2 Hz) and 0.2 G acceleration.

Figure 11 shows the maximum output power as a function of load resistance value. The output power represents the power consumption at the load resistance when the load resistance value is varied from 1 to 31 MΩ. Here, the result is measured under the resonance condition for each harvester. From the resistance value response of the output power, the optimum load resistance is 17 MΩ for the meshed-core PVEH and 13 MΩ for the solid-core type. These measured load resistance values are relatively high compared to those of the vibration energy harvesters using PZT or barium titanate as the piezoelectric material. The first reason for the high optimum load resistance values is the low resonance frequency such as about 20 Hz. Second is the lowered total capacitance by connecting the piezoelectric films in series. Third is the low relative permittivity of the piezoelectric PVDF film. Although the frequency depends on the external vibration and cannot be controlled, the optimum load resistance value can be reduced by connecting the PVDF films in parallel and improving the electrical properties of the material.

**Figure 12.**
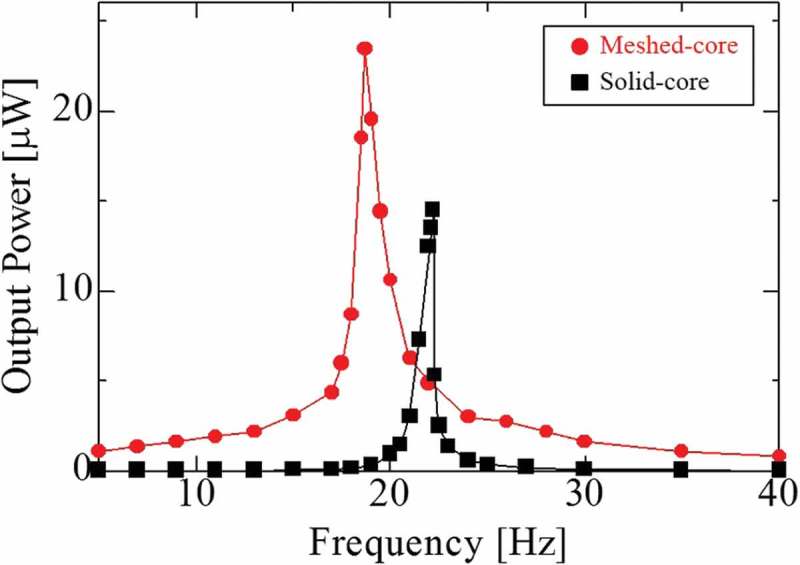
Maximum output power as a function of vibration frequency under each optimum load resistance (meshed-core 17 MΩ, solid-core 13 MΩ) and 0.2 G acceleration.

Figure 12 shows the frequency response from 1 to 40 Hz. In the meshed-core PVEH, the maximum output power is 24.6 μW, which is 68.5% higher than that of the solid-core type. This is because the bending stiffness of the bimorph cantilever is lowered by using the 3D meshed-core structure with voids. In other words, the amount of strain occurring in the piezoelectric film, as well as the amount of power generation, increases as the tip deflection increases due to the low bending stiffness. Furthermore, the resonance frequency is 18.7 Hz for the meshed-core PVEH and 22.2 Hz for the solid-core type. Here, the meshed-core type shows 15.8% lower resonance frequency than the solid-core type due to the low bending stiffness of the meshed-core structure. Besides the flexible characteristics of the 3D meshed-core elastic layer, both elastic layer and piezoelectric films are made of a polymer material. This is why the low resonance frequency, under 20 Hz, can be realized even in a small bimorph-type vibration energy harvester. Here, these experimental results – the results of output power and resonance frequency – agree with the value and tendency of the piezoelectric coupling analysis result. The cause of the errors between the experimental and FEM analysis is the difference in the detailed physical, mechanical, and piezoelectric parameters. For a better agreement with the results, it is necessary to measure and set the parameters more precisely.

Table 3 presents the measured results and calculated values under resonance condition, including the effective voltage, average power, the effective voltage, average power, and tip deflection. Here, the resonance frequency is lower compared with other piezoelectric cantilever-type vibration energy harvesters [4,5,27].

**Table 3.** Experimental results of meshed- and solid-core PVEHs.

	Meshed-core	Solid-core
Resonance frequency [Hz]	18.7	22.2
Effective voltage of load resistance (RMS) [V]	14.4	10.1
Average output power [μW]	12.3	7.3
Tip deflection [mm]	6.0	4.6

In summary, the performance of the bimorph-type vibration energy harvester is improved by the flexible 3D meshed-core structure of the elastic layer without changing the composed material, harvester size, and the basic fabrication process. Therefore, the approach for making the elastic layer flexible is effective for the bimorph-type PVEHs. Moreover, as reported in previous studies [19–21], the output power can be further improved by optimizing the shape and size and the resonance frequency can be further decreased by adjusting the weight of the proof mass.

Furthermore, to obtain high output power at low frequencies, it is necessary to improve both piezoelectric constant and the electromechanical coupling coefficient of flexible piezoelectric polymer materials. In the proposed method, the flexibility of the elastic layer is adjusted by 3D microstructure, and the limit in the device design depends on the flexibility of the piezoelectric material. In addition, when the flexibility of the piezoelectric material becomes higher than the flexibility of the elastic layer, the neutral axis enters the piezoelectric layer and the strain amount decreases in commonly studied unimorph-type PVEHs. Therefore, if more flexible piezoelectric materials are obtained, it is possible to further enhance the performance of the proposed method. Moreover, the piezoelectric constant of piezoelectric polymer such as PVDF is several tens of pC/N, which is 5 to 10 times inferior to inorganic ceramic materials [28,29]. Also, the electromechanical coupling coefficient of piezoelectric polymer is as small as five times that of inorganic ceramics [28,29]. Thus, improvement of these material properties is desired. In addition to that, it is desirable to reduce the cost of materials and fabrication process for the realization of IoT services. If these issues could be solved, the number of practical low-frequency vibration energy harvesters would increase.

## Conclusions

In this study, we confirmed the validity of the bimorph PVEH where a 3D meshed-core structure acts as an elastic layer. First, in the FEM analysis section, we demonstrated that the bending stiffness can be adjusted by changing the line spacing of meshed-core structure. Second, we showed the fabrication process of the 3D meshed-core PVEH by inclined photolithography and a simple bonding process. According to vibration tests, the resonance frequency of the meshed-core PVEH was 26% lower and the tip deflection was 1.3 times larger as compared to the solid-core PVEH. The maximum attained electrical output, 24.6 µW, is suitable for powering sensor nodes in wireless sensor networks. In conclusion, in this paper, we proposed a method for improving the output power and lowering the resonance frequency without any change in the material, device size, and basic fabrication process.
